# Phylogenetic analysis based on single-copy orthologous proteins in highly variable chloroplast genomes of *Corydalis*

**DOI:** 10.1038/s41598-022-17721-y

**Published:** 2022-08-20

**Authors:** Xianmei Yin, Feng Huang, Xiaofen Liu, Jiachen Guo, Ning Cui, Conglian Liang, Yan Lian, Jingjing Deng, Hao Wu, Hongxiang Yin, Guihua Jiang

**Affiliations:** 1 College of Pharmacy, Chengdu University of Traditional Chinese Medicine, Chendu, 611130 China; 2 Central Laboratory, Shandong Academy of Chinese Medicine, Jinan, 250014 China; 3 College of Pharmacy, Shandong University of Traditional Chinese Medicine, Jinan, 250355 China

**Keywords:** Phylogenetics, Genome

## Abstract

*Corydalis* is one of the few lineages that have been reported to have extensive large-scale chloroplast genome (cp-genome) rearrangements. In this study, novel cp-genome rearrangements of *Corydalis pinnata*, *C. mucronate*, and *C. sheareri* are described. *C. pinnata* is a narrow endemic species only distributed at Qingcheng Mountain in southwest China. Two independent relocations of the same four genes (*trnM-CAU-rbcL*) were found relocated from the typically posterior part of the large single-copy region to the front of it. A uniform inversion of an 11–14-kb segment (*ndhB-trnR-ACG*) was found in the inverted repeat region; and extensive losses of *accD*, *clpP,* and *trnV-UAC* genes were detected in all cp-genomes of all three species of *Corydalis*. In addition, a phylogenetic tree was reconstructed based on 31 single-copy orthologous proteins in 27 cp-genomes. This study provides insights into the evolution of cp-genomes throughout the genus *Corydalis* and also provides a reference for further studies on the taxonomy, identification, phylogeny, and genetic transformation of other lineages with extensive rearrangements in cp-genomes.

## Introduction

*Corydalis* DC. is a large and diverse genus, with ~ 786 species, within the family Papaveraceae (http://www.worldfloraonline.org/downloadData [accessed 9 December 2021]). Plants belonging to the genus *Corydalis* are distributed in the Hengduan Mountains and Qinghai–Tibet Plateau and adjacent areas^1^. The structures of the some recognized *Corydalis* chloroplast genomes (cp-genomes) have undergone a series of genetic rearrangements, such as pseudogenization or the loss of genes, to adapt to drastic changes in the environment^1–4^. *Corydalis pinnata* is a narrow endemic species in China and is only distributed along the streams of Qingcheng Mountain in southwest China at altitudes between 1300 m a.s.l. and 1400 m a.s.l. Consequently, this species must also have undergone a unique genetic shift.

Most of the *Corydalis* plants have potential as medicinal agents due to their therapeutic effects against hepatitis, tumors, cardiovascular diseases, and pain^5,6^, but some species are toxic^7^. As one of the most taxonomically challenging plant taxa, the genus *Corydalis* has extremely complex morphological variations because of typical reticulate evolution and intense differentiation during evolution^8^, which has hampered understanding of the identification, taxonomy, and utilization of members of this genus.

Chloroplasts are common organelles with an essential role in the photosynthesis of green plants^9^. The cp-genome is an ideal research model for studying molecular identification, phylogeny, species conservation, and genome evolution because of its conservative structure^10,11^. The increasingly wide application of the cp-genome super-barcode in identification make the development of new cp-genome resources urgent and significant^12,13^. Cp-genome rearrangements can also be useful as a phylogenetic marker because they lack homoplasy and are easily identified^14–16^. Although some genetic rearrangements of *Corydalis* cp-genomes have been reported^1,2^, the pattern, origin, evolution, and phylogenetic relationship of cp-genome rearrangements in *Corydalis* remain unclear because of a lack of sufficient genetic resources. In the present study, three species of the genus *Corydalis* from Qingcheng Mountain, including a narrow endemic species, were identified based on their cp-genomes. In addition, 12 *Corydalis* cp-genomes from the National Centre for Biotechnology Information (NCBI) database were included in the rearrangement analysis to represent all five subgenera of *Corydalis* and cover most of the distribution areas*.* The structural characteristics, repeat sequences, and cp-genome rearrangements were documented, and phylogenetic trees based on single-copy orthologous proteins were analyzed. The aim of the study was to assess structural variation and provide valuable resources for identification and classification of members of the genus *Corydalis*.

## Results

### DNA features of three *Corydalis cp-genomes*

**Figure 1:**
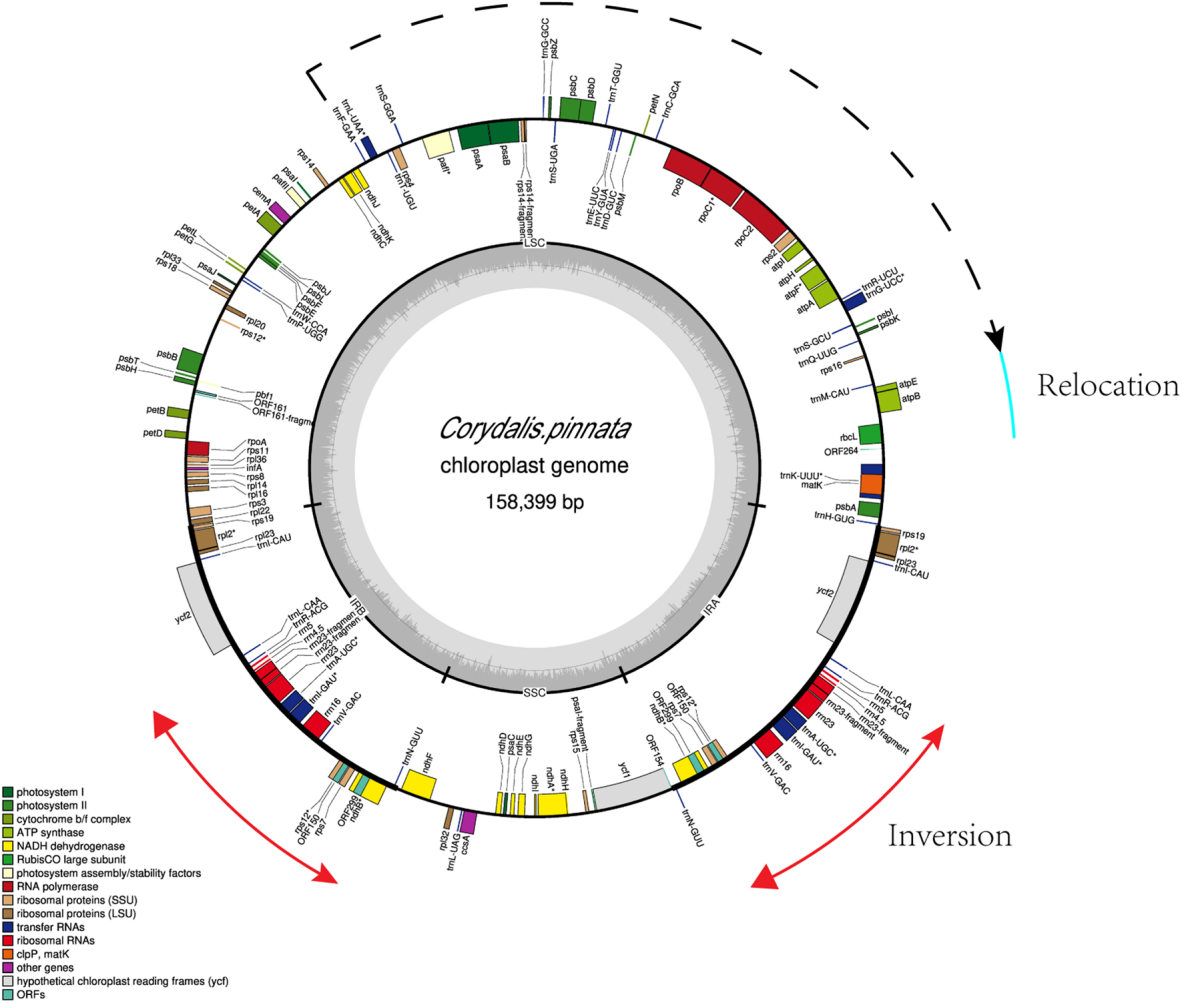
Gene map of the chloroplast genome of *C. pinnata*. Genes within the circle are transcribed clockwise, and those outside are transcribed counterclockwise. Genes belonging to different functional groups are colour coded. The dark grey in the inner circle corresponds to DNA G+C content, and the light grey corresponds to A+T content.

Cp-genomes of three species of the genus *Corydalis* were sequenced; the three species were *Corydalis pinnata*, *C. mucronate,* and *C. sheareri*. The sizes of the three newly sequenced *Corydalis* cp-genomes ranged from 158,399 bp (*C*. *pinnata*) to 161,105 bp (*C. sheareri*) (Table 1). The guanine+cytosine (G+C) contents of the three genomes were 39.6%–40.47%. The three species each had a cp-genome with typical angiosperm quadripartite structure: a large single-copy (LSC) region, a small single-copy (SSC) region, and a pair of inverted repeats (IRs: IRA and IRB). The lengths of the LSC, SSC, and IR regions of the three newly sequenced *Corydalis* cp-genomes were 87,573–90,438, 20,408–23,322 and 23,778–25,209 bp, respectively (Table 1). After annotation, the sequences of the whole cp-genome sequences of the three *Corydalis* plants were submitted to the NCBI database; GenBank accession numbers are supplied in Table 1. The *C. pinnata* cp-genome was taken as an example and a physical map of the cp-genome was created according to the annotation results using OrganellarGenomeDRAW (OGDraw)^17^ (Fig. 1). A total of 115–117 unique genes, comprising 80–83 protein-coding genes, 28–30 tRNA genes, 4 rRNA genes, and 4–6 pseudogenes, were present in the three newly sequenced *Corydalis* cp-genomes (Table 1). In total, seven genes were pseudogenized in one or more *Corydalis* cp-genomes, and three genes (*accD*, *clpP*, and *trnV-UAC*) were lost in the three newly sequenced *Corydalis* cp-genomes (Supplementary Table 1).

**Table 1:** Summary of genome structure, and gene content of the three newly sequenced Corydalis cp-genomes.

	Total reads	Total base	Q30	GC	Genome size(bp)	LSC	IR	SSC	Coding gene	tRNA	rRNA	Pseudogene	Assession number
*C. pinnata*	39,260,005	11.72	88.3	40.47%	158,399	87,573	25,209	20,408	83	28	4	4	OK647837
*C. mucronata* Franch	34,882,414	10.42	88.11	40.44%	159,803	88,925	23,778	23,322	82	29	4	5	OK647838
*C. sheareri* S. Moore	40,305,430	12.07	86.86	39.60%	161,105	90,438	23,788	23,091	80	30	4	6	OK647839

In contrast to previously reported *Corydalis* cp-genomes^1^, the three newly sequenced *Corydalis* cp-genomes in this study had 11 complete *ndh* genes (Supplementary Table 1). In addition, amongst all the anticipated genes of the three *Corydalis* cp-genomes, introns were discovered in 11–13 genes, including 4–5 tRNA genes and 7–8 protein-coding genes (Supplementary Table 2). The tRNA genes with introns were *trnL-UAA*, *trnK-UUU*, *trnI-GAU*, *trnG-UCC*, and *trnA-UGC*. The eight protein-coding genes with introns were *rps12*, *rpoC1*, *rpl2*, *pafI*, *ndhB*, *ndhA*, *atpF*, and *ycf2*. Two of the 13 intron-containing genes had two introns (*rps12* and *pafI*); the remainder of the genes contained only one intron. The *trnH-UUU* gene contained the largest intron (2474–2488 bp), which contained the whole *matK gene*. Similar to other angiosperms, the gene *rpl2* in the three *Corydalis* cp-genomes resulted from trans-splicing activity. The 5ʹ end of *rpl2* lay in the LSC region, and the 3ʹ end was located in the IR region (Supplementary Table 2).

### Chloroplast genome structure rearrangement

**Figure 2:**
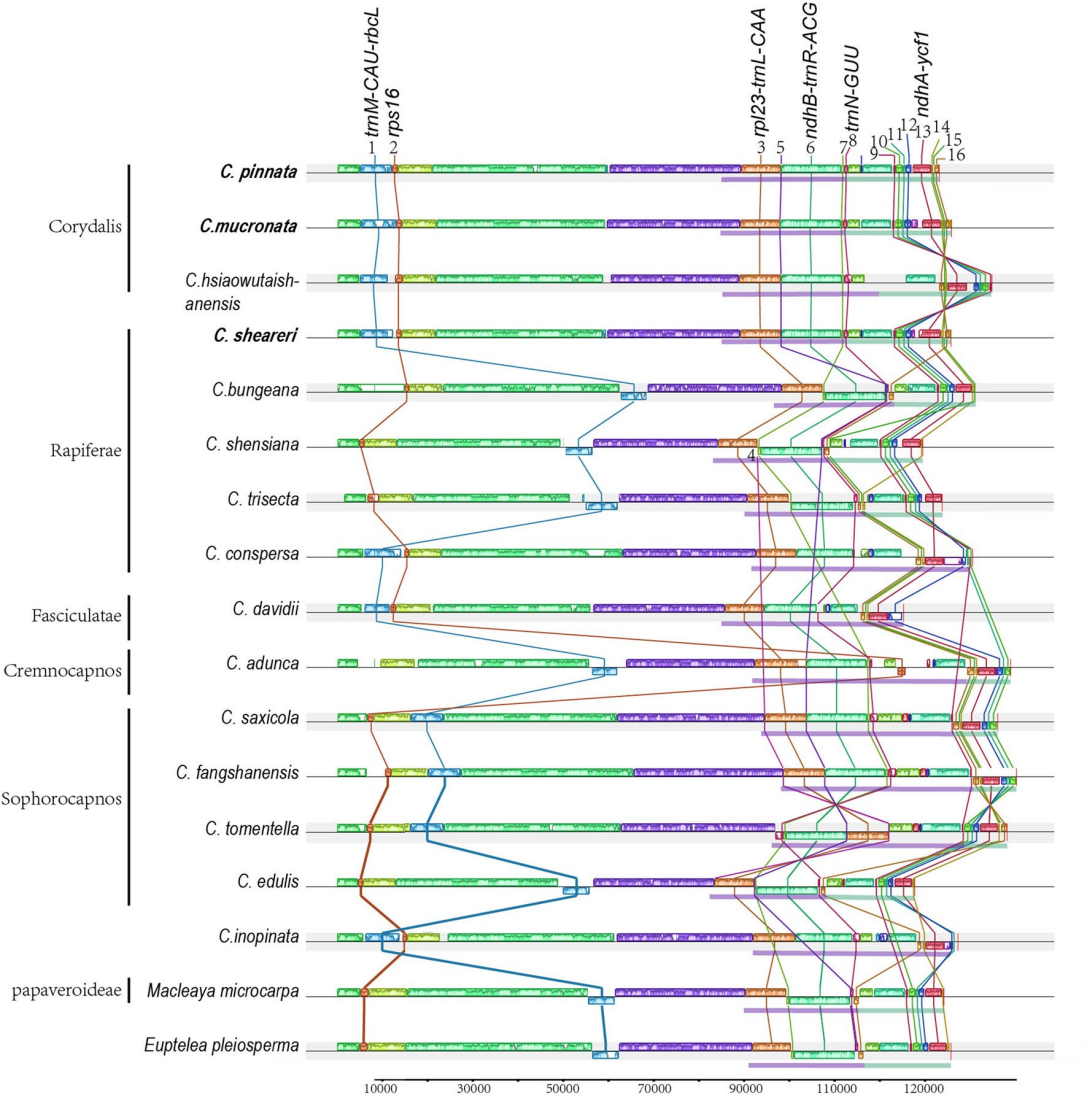
Chloroplast structural alignment of *Corydalis* and *E. pleiosperma* using Mauve. LCBs are represented by coloured blocks, and the blocks connected by lines indicate homology. Violet lines below each cp-genome represent IR regions, and green lines represent SSR regions. Species in bold are newly sequenced.

Seventeen cp-genomes were included in the syntenic comparisons by Mauve alignment (Fig. 2), including 15 *Corydalis* cp-genomes, a representative of Papaveroideae cp-genomes (*Macleaya microcarpa*), and a sister in the Ranunculales cp-genomes (*Euptelea pleiosperma*) to represent a typical angiosperm quadripartite cp-genome structure. More than 30 locally collinear blocks (LCBs) were identified in the *Corydalis* cp-genomes, from which 15 rearrangements were deduced (Fig. 2).

A total of 16 relocation blocks were identified in the 15 *Corydalis* cp-genomes. Block 1 (approximately 6 kb) of 10 *Corydalis* cp-genomes contained 4–5 genes (*trnM-CAU*, *atpE*, *atpB*, *rbcL*, and *trnV-UAC*) relocated from the classically posterior part of the LSC region (downstream of the *ndhC* gene) to the front. Of these cp-genomes with block 1, three cp-genomes from subgenus *Sophorocapnos* (*C. saxicola*, *C. fangshanensis*, and *C. tomentella*) displayed different types of relocation (downstream of the *atpH* gene) from other subgenera (downstream of the *trnK-UUU* gene). Then, in the cp-genome of *C. adunca* (subg. *Cremnocapnos*), block 2 with 1 kb of the *rps16* gene relocated from the typical LSC region to downstream of the *ndhF* gene in the IR region. In addition, blocks 5–7 with approximately 13 kb in the IR region contained 11 genes (*ndhB-trnR-ACG*) inverted uniformly in *C. pinnata*, *C. mucronata*, *C. hsiaowutaishanensis* (subg. *Corydalis*), *C. sheareri* (subg. *Rapiferae*), *C. adunca* (subg. *Cremnocapnos*), *C. saxicola*, and *C. fangshanensis*. In *C. conspersa* (subg. *Rapiferae*) and *C. davidii* (subg. *Fasciculatae*), block 6 was also inverted but blocks 5 and 7 were lost. Blocks 12–15 (~ 8 kb) in the SSC region contained five genes (*ndhA-ycf1*) inverted uniformly in *C. hsiaowutaishanensis*, *C. conspersa*, *C. davidii*, *C. adunca*, *C. saxicola*, and *C. inopinata.* Moreover, blocks 9–11 were inverted with blocks 12–15 in *C. hsiaowutaishanensis*, *C. conspersa*, and *C. inopinata*, whereas blocks 9–11 and 16 underwent various degrees of loss in *C. davidii*, *C. adunca*, *C. saxicola*, and *C. fangshanensis*. In addition, blocks 3–8, with approximately 36 kb in the IR region, contained 54 genes (*trnN-GUU-psaI*) and were inverted uniformly in *C. tomentella* (subg. *Sophorocapnos*) compared with *C. fangshanensis* and *C. tomentella* from the same subgenus.

### Comparison of genomic variation in the three newly sequenced *Corydalis cp-genomes* and *C. edulis* cp-genome

**Figure 3:**
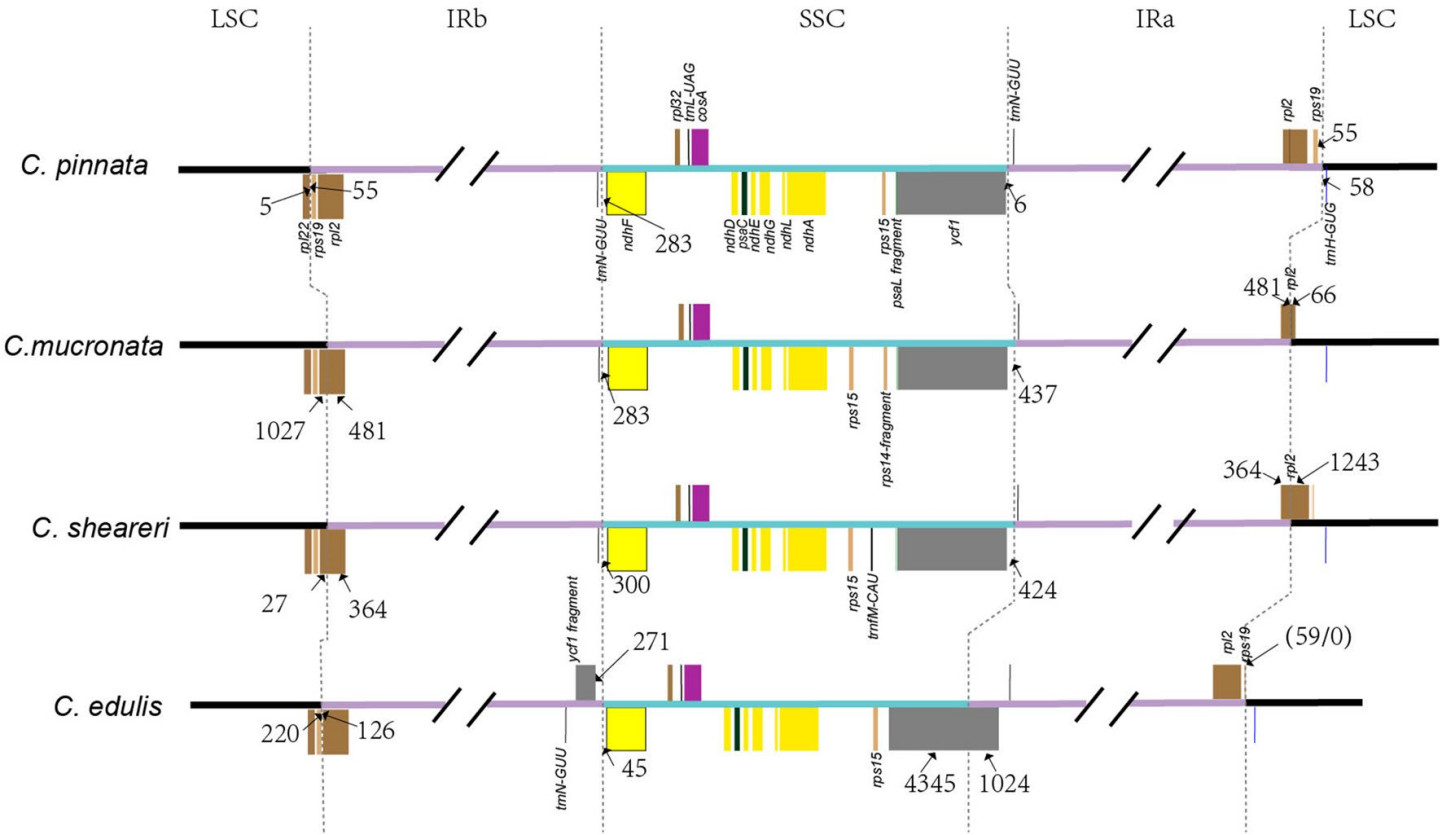
Comparison of genome boundaries in chloroplasts from *C. pinnata*, *C. mucronata*, *C. sheareri* and *C. edulis.*

Previous studies reported a marked IR region expansion in some *Corydalis* cp-genomes; the IR region expanded into the simple sequence repeat (SSR) region and led to IR–SSC boundary variations^1,2^. In the present study, three newly sequenced *Corydalis* cp-genomes were compared with the *C. edulis* cp-genome, which exhibited a typical angiosperm quadripartite cp-genome structure (Fig. 3). The location of the IR region in the three newly sequenced *Corydalis* cp-genomes was relatively conservative (Fig. 3). In these three species, *rps19* was located in the LSC region, and *ndhF* was in the SSC region. The coding region of *rpl2* was in the IR region of the *C. pinnata* cp-genome but spanned the LSC and IRa regions of the *C. mucronata* and *C. sheareri* cp-genomes; therefore, the IRb/LSC boundary (the 5′ end was lost) region created a pseudogene.

**Figure 4:**
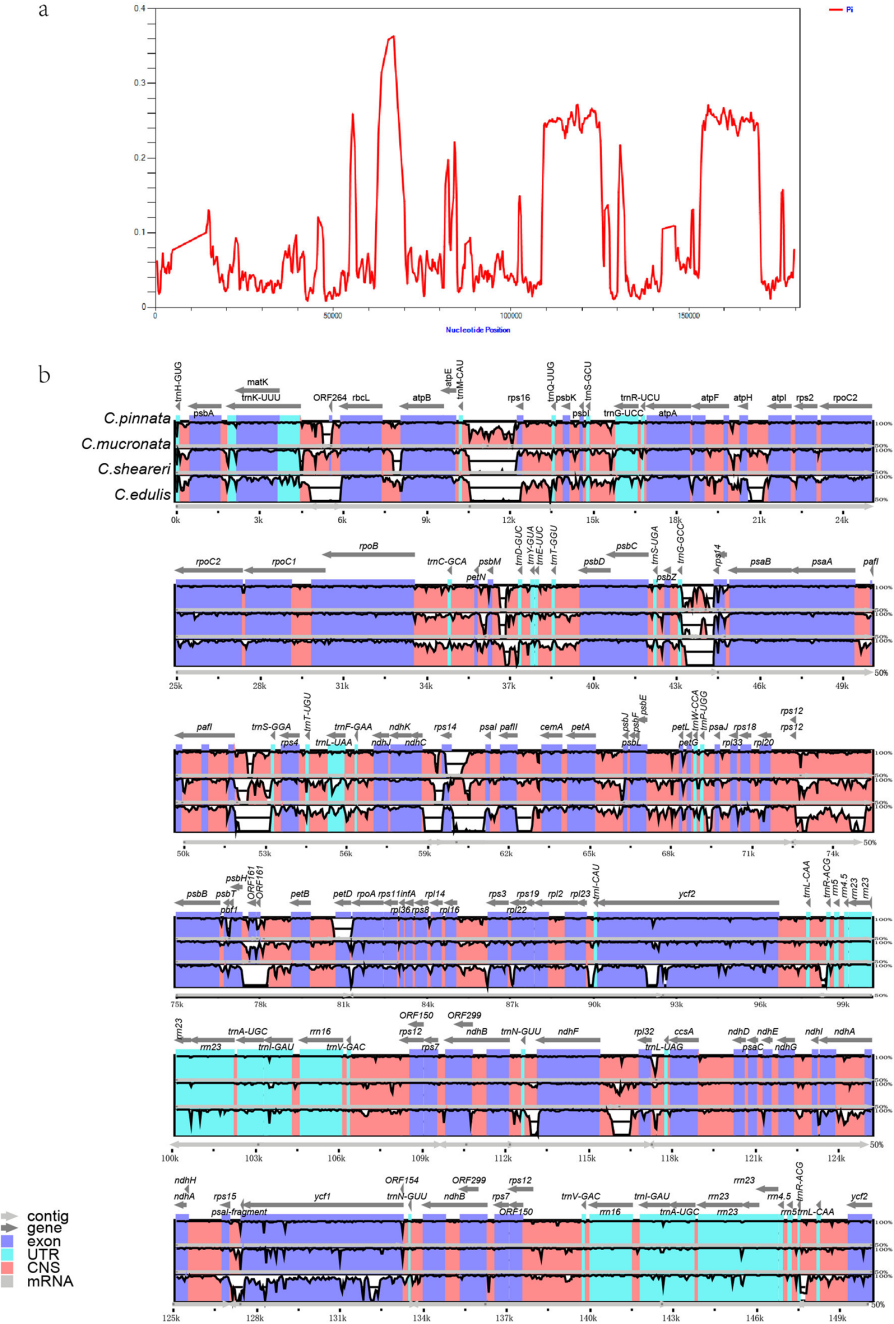
Comparative analyses of genomic differences in the chloroplasts of *C. pinnata*, *C. mucronata*, *C. sheareri* and *C. edulis*. (**a**), Sliding window analyses of the entire cp-genome. (**b**), Alignment visualisation of the chloroplast genome sequences of *C. pinnata*, *C. mucronata*, *C. sheareri* and *C. edulis* using mVISTA. Grey arrows and thick black lines indicate genes and their orientation. Purple bars indicate exons, blue bars represent untranslated regions, pink bars represent non-coding sequences, and grey bars denote mRNA. The similarity among the chloroplast genomes is shown on a vertical scale ranging from 50 to 100%.

The *C. edulis* cp-genome was used as a reference to ascertain differences in the genomic sequences of the three newly sequenced *Corydalis* cp-genomes (Fig. 4a,b). The rearranged regions exhibited higher variability compared with the other regions of the four *Corydalis* cp-genomes studied (Fig. 4a). Similar to other cp-genomes of angiosperms, most of the protein-coding genes were highly conserved, except for the large variation in the protein-coding genes of some genes (e.g., *rps19*, *rpl22*, *ycf1* and *ycf2*), intron regions (*paf1*, *ndhA* and *rpl2*), and intergenic regions (*trnQ-UUG-psbK*, *psbK-psbI*, *atpF-aptH*, *atpH-atpI*, *rpoB-trnC-GCA*, *trnC-GCA-petN*, *trnT-GGU-psbD*, *trnE-UUC-trnT-GGU*, *trnD-GUC-trnY-GUA*, *psaA-pafI*, *pafI-trnS-GGA*, *rps4-trnT-UGU*, *trnT-UGU-trnL-UAA*, *trnR-ACG-trnL-CAA*, and *trnN-GUU-ndhB*) among the chloroplast genomic sequences with a higher degree of variation. Such higher-resolution loci have the potential to be used as barcodes in species identification.

### Analyses of long repetitive sequences and SSRs

**Figure 5:**
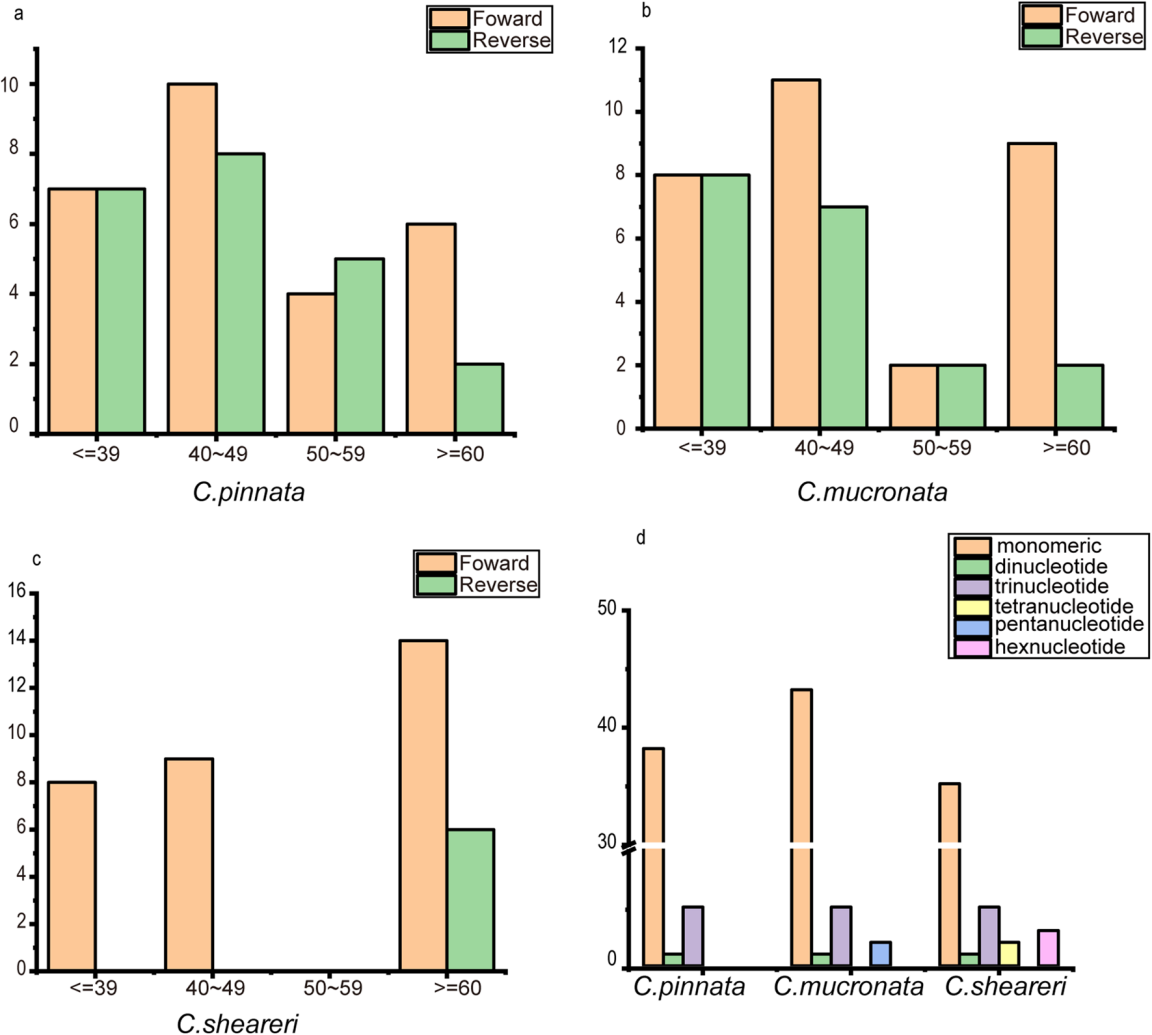
Long repetitive sequences and SSR distribution in the chloroplast genomes of *C. pinnata*, *C. mucronata* and *C. sheareri.*

Interspersed repeated sequences (IRSs) with a repeat unit length of ≥ 39 bp were evaluated in the chloroplast genomes of *C. pinnata*, *C. mucronate*, and *C. sheareri*. These repeats comprised only forward and palindromic repeats and lacked reverse and complementary repeats that are common in other species. Fifty IRSs were found, and among these, the sequence lengths in *C. pinnata, C. mucronate*, and *C. sheareri* were 40–49, > 80, and ≤ 49/≥ 80 bp, respectively*.* The IRS analyses of the chloroplast genomes are shown in Fig. 5a–c.

In total, 46 SSRs were found in *C. pinnata*, including 38 mononucleotide repeats, 1 dinucleotide repeat, and 5 trinucleotide repeats: 51 SSRs were identified in *C. mucronate*, including 43 mononucleotide repeats, 1 dinucleotide repeat, 5 trinucleotide repeats, and 2 pentanucleotide repeats; and 46 SSRs were found in *C. sheareri,* including 35 mononucleotide repeats, 1 dinucleotide repeat, 5 trinucleotide repeats, 2 tetranucleotide repeats, and 3 hexanucleotides (Fig. 5d).

### Phylogenetic analyses

**Figure 6:**
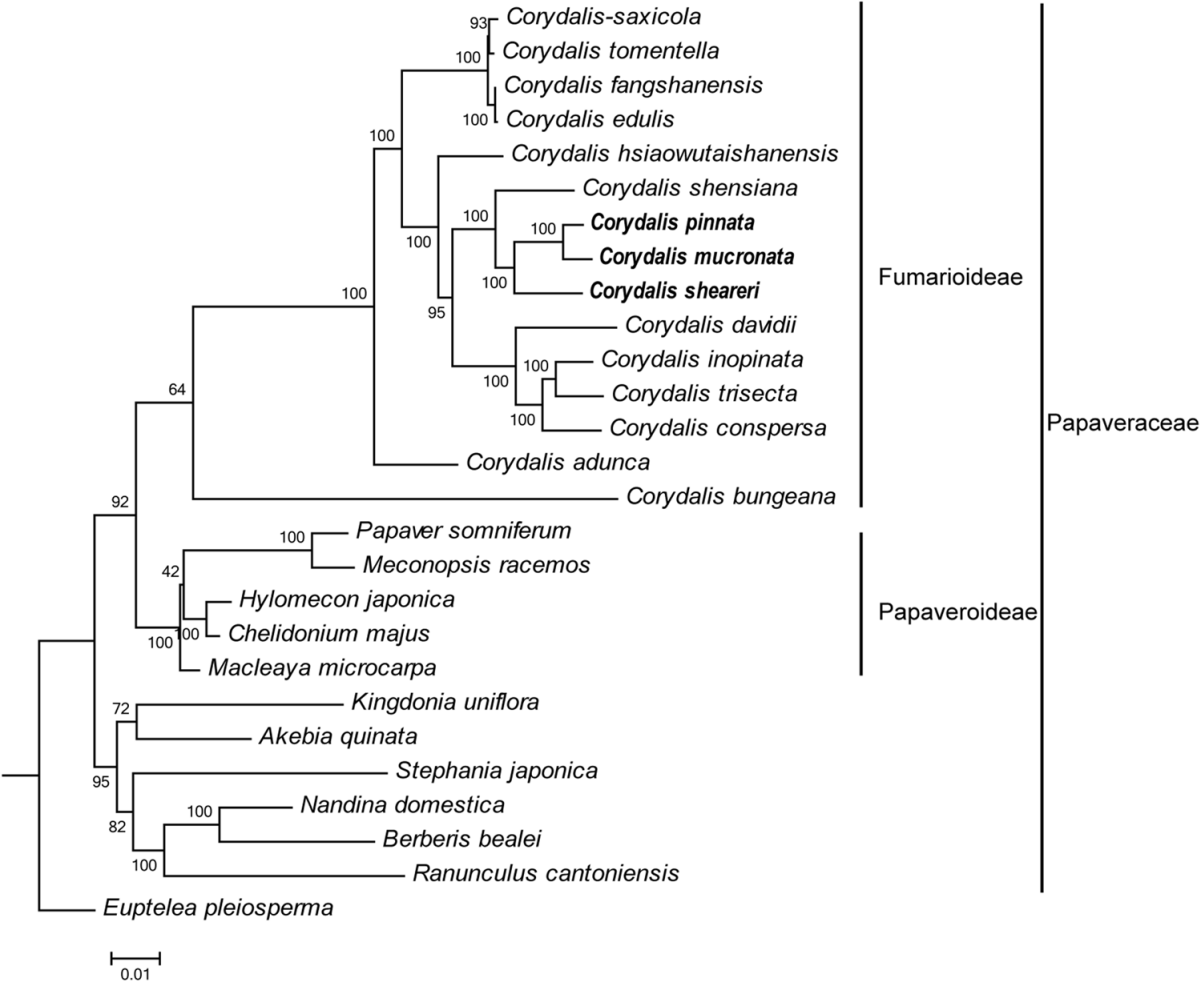
ML tree of *Corydalis* and its relative species based on single-copy orthologous proteins. *Euptelea pleiosperma* was used as outgroup. References for the classification of the genus *Corydalis* were World Flora Online. Bootstrap supports on the branches were calculated from 1000 replicates. Three newly sequenced *Corydalis* marked with bold.

Using concatenated single-copy orthologous proteins to resolve phylogenic relationships could avoid rearrangement-misled phylogenetic tree reconstruction and provide a more reliable evolutionary framework compared with using several specific genes^18^. Therefore, the predicted proteome was used in the phylogenetic analyses rather than the whole cp-genome sequence. Based on 31 single-copy orthologous proteins conserved in 27 species with *E. pleiosperma* as the outgroup, a maximum-likelihood (ML) phylogenetic tree was reconstructed to illuminate the evolutionary history of the compared species (Fig. 6). The ML tree had three major clades: the Fumarioideae clade, Papaveroideae clade, and the clade with the rest of the Ranunculales family members. *Corydalis* constituted a monophyletic sub-clade nested within the Fumarioideae clade. All lineages within *Corydalis* were strongly supported. The three newly sequenced *Corydalis* cp-genomes, namely, *C. pinnata* (Sect. Mucronatae), *C. mucronata* (Sect. Mucronatae), and *C. sheareri* (Sect. Asterostigmata), were closely related.

## Discussion

Although the three newly sequenced *Corydalis* cp-genomes from the same geographic region belong to two different subgenera of *Corydalis*, the sizes and structures of their LSC, IR, and SSC regions, as well as their total genomes, are highly similar. This includes similar gene losses, inversions, and relocations (Fig. 1 and Supplementary Table 1), which are common features in the *Corydalis* cp-genomes and are considered to be responsible for the variation in cp-genome sizes^1^.

The loss of three genes (*accD*, *clpP*, and *trnV-UAC*) is a synapomorphic characteristic in the *Corydalis* cp-genomes (Supplementary Table 1). Xu et al.^1^ speculated that the loss of the *accD* gene occurred before divergence of the genus *Corydalis*. However, in the present study, the *accD* gene was found in the cp-genomes of a few species of the subgenus Rapiferae (Supplementary Table 1), which indicated that the loss event happened after divergence of the genus *Corydalis*. The exact time of the loss event should be further explored by gathering more information on *Corydalis* cp-genomes. The *accD* gene is relocated to the nucleus in some species, such as some members of the family Campanulaceae^19,20^. The pseudogenization or loss of 11 chloroplast *ndh* genes that encode NADH dehydrogenase subunits only occurred in a few species of the genus *Corydalis* (*C. conspersa*, *C. davidii*, *C. adunca*, and *C. inopinata*; Supplementary Table 1). Strikingly, these species are all located in high-altitude areas (1000–5200 m a.s.l.)^21^. Therefore, extreme changes in the environment may result in gene deletions or pseudogenization; this phenomenon has been observed in other species^22^. Further studies are required to determine whether or not the pseudogenization or loss of *ndh* genes will affect photosynthesis in those plants.

The chloroplast genome, as a photosynthetic organelle, is highly conserved in terms of structure, gene content, and arrangement^23–25^. Large-scale rearrangement exists only occasionally in a few lineages, such as Campanulaceae^16,17,26–28^, Ranunculaceae^29,30^, Geraniaceae^31–36^, Fabaceae^15,37–44^, Oleaceae^45^, Asteraceae^46–49^, Plantaginaceae^50–52^, Euphorbiaceae^53^ and Poaceae^14,54–57^. In the present study, rearrangement predominantly occurred in 16 regions (blocks 1–16, Fig. 2) of *Corydalis* plants, which determine the diversity in *Corydalis* cp-genomes. Repeat sequences may contribute to structural variations in relatively stable rearrangement regions^58–60^. Relocation only occurred in the LSC region of the *Corydalis* cp-genomes, and inversion only occurred in the IR and SSC regions (Fig. 2). This suggested that the patterns of relocation and inversion were regulated in different ways. In addition, blocks 1–16 are likely active rearrangement regions because they have various rearrangement patterns. *C. hsiaowutaishanensis* (subg. *Corydalis*), *C. adunca* (subg. *Cremnocapnos*), *C. Saxicola*, and *C. fangshanensis* (subg. *Sophorocapnos*) all underwent the inversion of blocks 10–16, but the inversion boundaries of *C. hsiaowutaishanensis* expanded into block 9, suggesting that the inversion of blocks 9–16 in *C. hsiaowutaishanensis* was an independent event. Furthermore, some species from different subgenera have the same relocation or inversion pattern, such as the three *Corydalis* plants (*C. pinnata*, *C. mucronate*, and *C. sheareri*) collected from Qingcheng Mountain in the current study. Although they represent two subgenera, these three species have an almost identical relocation/inversion pattern in their cp-genomes (Fig. 2). Moreover, blocks 5–7 underwent at least two inversions in *C. tomentella*; blocks 5–7 initially inversed independently and then inversed with blocks 3, 4, and 8. This active rearrangement suggested that relocation or inversion in *Corydalis* cp-genomes might be affected by the geographical environment.

Loss of introns and/or genes is instrumental in the regulation of gene expression and can control gene expression temporally and in a tissue-specific manner^61–63^.The regulation mechanisms of introns for gene expression in plants and animals have been reported^63–65^. However, the implications or link between gene expression and intron loss for *Corydalis* have not been published. Further experimental work on the roles of introns in *Corydalis* is therefore essential and should prove interesting. Highly variable DNA barcodes play an important role in species identification and phylogenetic analyses. In the current study, protein-coding genes (*rps19*, *rpl22*, ycf1, and *ycf2*), intron regions (*paf1*, *ndhA*, and *rpl2*), and the intergenic regions (*trnQ-UUG-psbK*, *psbK-psbI*, *atpF-aptH*, *atpH-atpI*, *rpoB-trnC-GCA*, *trnC-GCA-petN*, *trnT-GGU-psbD*, *trnE-UUC-trnT-GGU*, *trnD-GUC-trnY-GUA*, *psaA-pafI*, *pafI-trnS-GGA*, *rps4-trnT-UGU*, *trnT-UGU-trnL-UAA*, *trnR-ACG-trnL-CAA*, and *trnN-GUU-ndhB*) exhibited some extent of variation and have great potential as DNA markers (Fig. 4b).

Cp-genomes have made marked contributions to the phylogenetic studies of angiosperms and to resolving the evolutionary relationships within phylogenetic clades^66,67^. However, active rearrangement in *Corydalis* cp-genomes may mislead the reconstruction of species phylogenetic relationships based on DNA sequence of cp-genomes. Phylogenetic reconstruction of the genus *Corydalis* was previously explored with DNA barcoding^68^ or relatively conserved nucleotide fragments in cp-genomes^1^. However, deep relationships remained poorly resolved by this phylogenetic approach applying a few plastid markers. Some studies reported that the protein-coding genes shared by all taxa could be used to reconstruct a phylogeny^2,34^. However, single-copy genes (SCGs) have subsequently emerged as candidates for phylogenetic analysis because paralogues are derived from duplication events other than speciation events and should therefore be discarded from phylogenetic analyses^69,70^. Therefore, the 31 single-copy orthologous proteins in all 27 cp-genomes were used to reconstruct the phylogeny of the genus *Corydalis.* Three distinct clades were defined by high bootstrap values (Fig. 6) in the resulting phylogenetic tree, which is consistent with previous studies based on molecular markers^1,71^. This indicated that the application of the single-copy orthologous proteins of cp-genomes can improve the resolution of the phylogeny and taxonomy of the genus *Corydalis*. Findings from the study also provide a reference for the taxonomy and identification of other plants with extensive rearrangement in cp-genomes.

## Conclusions

The cp-genomes of three species of the genus *Corydalis* (*C. pinnata*, *C. mucronata*, and *C. sheareri*) from the Qingcheng Mountain in southwest China, including a narrow endemic species (*C. pinnata*), were characterized. The cp-genomes of the three species exhibited a large-scale rearrangement, including the relocation of four genes (*trnM-CAU-rbcL*) in the LSC region, the inversion of an 11–14-kb segment (*ndhB-trnR-ACG*) in the IR region, and the loss of three genes (*accD*, *clpP*, and *trnV-UAC*). The three *Corydalis* cp-genomes showed high similarity in terms of genome size, gene classes, gene sequences, rearrangement pattern, and distribution of repeat sequences. In addition, the structural alignment of 17 *Corydalis* cp-genomes with the typical chloroplast genomic structure of angiosperms (*E. pleiosperma*) revealed a frequent and extensive large-scale rearrangement in the *Corydalis* cp-genomes. Among them, the relocation of two blocks (*trnM-CAU-rbcL* and *rps16*) frequently appeared in the LSC region, and the inversion of four blocks (*rpl23-trnL-CAA*, *ndhB-trnR-ACG*, *trnN-GUU*, and *ndhA-ycf1*) frequently appeared in the IR and SSC regions. The extensive large-scale cp-genome rearrangement may mislead phylogenetic analysis based on cp-genomes. Single-copy orthologous proteins of cp-genomes were therefore used to reconstruct the phylogeny of the genus *Corydalis*. This method was concluded to have good prospects for elucidating the phylogeny and taxonomy of *Corydalis* and could potentially be employed for the phylogenetic analysis of other lineages with extensive rearranged cp-genomes in future studies. Findings from this study provide a reference for further studies on the taxonomy, identification, and evolution of the genus *Corydalis.*

## Materials and methods

### Plant collection and sampling

The aboveground parts of the three plant species were collected from Qingcheng Mountain, Sichuan Province, China (*C. sheareri*, location: E 103°32ʹ4″ N 30°54ʹ5″, altitude: 720 m a.s.l.; *C. mucronate*, location: E 103°28ʹ35″ N 30°28ʹ35″, altitude: 980 m a.s.l.; *C. pinnata*, location: E 103°25ʹ27″ N 30°65ʹ5″, altitude: 1350 m a.s.l.). The voucher specimens were deposited in the herbarium of the College of Pharmacy, Chengdu University of Traditional Chinese Medicine, China (deposition numbers: *C. sheareri*, CDCM0005283; *C. mucronate*, CDCM0005284; *C. pinnata*, CDCM0005285). The collection of samples conformed to the management provisions of the List of State-protected Wild Plants and was approved by the National Forestry and Grassland Administration of China (Supplementary Fig. 1). The specimens were identified by Professor Guihua Jiang.

### DNA sequencing, assembly and validation of the chloroplast genome

A modified cetyltrimethylammonium bromide (CTAB) method was used for DNA extraction and the NEBNext Ultra DNA Library Prep Kit for Illumina sequencing was used for 500-bp paired-end library construction. A shotgun library (250 bp) was constructed according to the manufacturer’s (Vazyme Biotech, Nanjing, China) instructions. Sequencing was accomplished with the X™ Ten platform (Illumina, San Diego, CA, USA) using the double terminal sequencing method (pair-end 150)^10^. Total raw data from a sample was approximately 10.0 G, and > 300 million paired-end reads were attained.

Raw data were filtered by Skewer-0.2.2 22^72^. The resulting reads were used for genome assembly by GetOrganelle version 1.7.5^73^. Another assembly for each species of the genus *Corydalis* was performed by ABYSS with *C. edulis* as the reference to confirm the GetOrganelle assemblies. The draft genome was used to map clean reads by BWA version 0.7.17^74^, and then clean reads were filtered using SAMtools version 1.7^75^. Mapping was visualized by IGV version 2.10.0^76^ to check the concatenation of contigs^1^. Furthermore, junction splicing sites were verified with polymerase chain reaction (PCR) and Sanger sequencing. All of the contigs were aligned to the reference cp-genome of *C. edulis* with MUMmer version 4.0^77^. Finally, the sequences were extended and gaps were filled with SSPACE-3.0^78^.

### Gene annotation and sequence analyses

Sequence annotation was achieved by Plann version 1.1.2^79^ using the cp-genome of *C. conspersa* as a reference and some manual correction*.* BLAST and Apollo^80^ were used to check the start and stop codons and the intron/exon boundaries with the cp-genome of *C. conspersa* as a reference sequence. Complete cp-genome sequences were submitted to the NCBI. A physical map of the cp-genomes was generated with Organellar Genome OGDraw^81^ (http://ogdraw.mpimp-golm.mpg.de/).

### Genome structure analyses

To determine synteny and identify possible rearrangements, 19 cp-genomes were compared using Mauve 2.4.0^82^ with the “progressiveMauve” algorithm, including 17 *Corydalis* cp-genomes, the cp-genome of *Macleaya microcarpa* (NC_039623*)* representing Papaveroideae, and the cp-genome of *Euptelea pleiosperma* (NC_029429) representing a typical angiosperm cp-genome. The Mauve result was then manually modified to show the notable rearrangements. The cp-genomes of species of the genus *Corydalis* were completed by mVISTA^83^ (Shuffle-LAGAN mode) using the genome of *C. edulis* as the reference. Tandem Repeats Finder^84^ was used to detect tandem repeats, forward repeats, and palindromic repeats as tested by REPuter^85^. SSRs were detected by Misa.pl^86^ using search parameters of mononucleotides set to ≥ 10 repeat units, dinucleotides ≥ 8 repeat units, trinucleotides and tetranucleotides ≥ 4 repeat units, and pentanucleotides and hexanucleotides ≥ 3 repeat units.

### Phylogenetic analyses

Twenty-seven cp-genomes were used to reconstruct a phylogenetic tree. First, single-copy orthologous proteins were extracted by OrthoFinder version 2.3.8^87^. Next, genes were aligned by MUSCLE version 3.8, and then the best-fit models of amino acid substitution were estimated by ProtTest version 3.4^88^ with the best corrected Akaike Information Criterion (AICc) value selected. Finally, a ML phylogenetic tree was reconstructed by RAxML version 8.2.12^89^ including tree robustness assessment using 1000 replicates of rapid bootstrap with the HIVb + I + G + F substitution model based on the results of ProtTest.

## Supplementary Information

Supplementary Figures.

Supplementary Tables.
