# Supplementary material for: Phylogenetic analysis based on single-copy orthologous proteins in highly variable chloroplast genomes of Corydalis
Source: Sci Rep. 2022 Aug 20;12:14241. doi: 10.1038/s41598-022-17721-y (PMC9392791; doi:10.1038/s41598-022-17721-y)
Supplement: Supplementary file 1 — Supplementary Figures. [file 41598_2022_17721_MOESM1_ESM.docx]

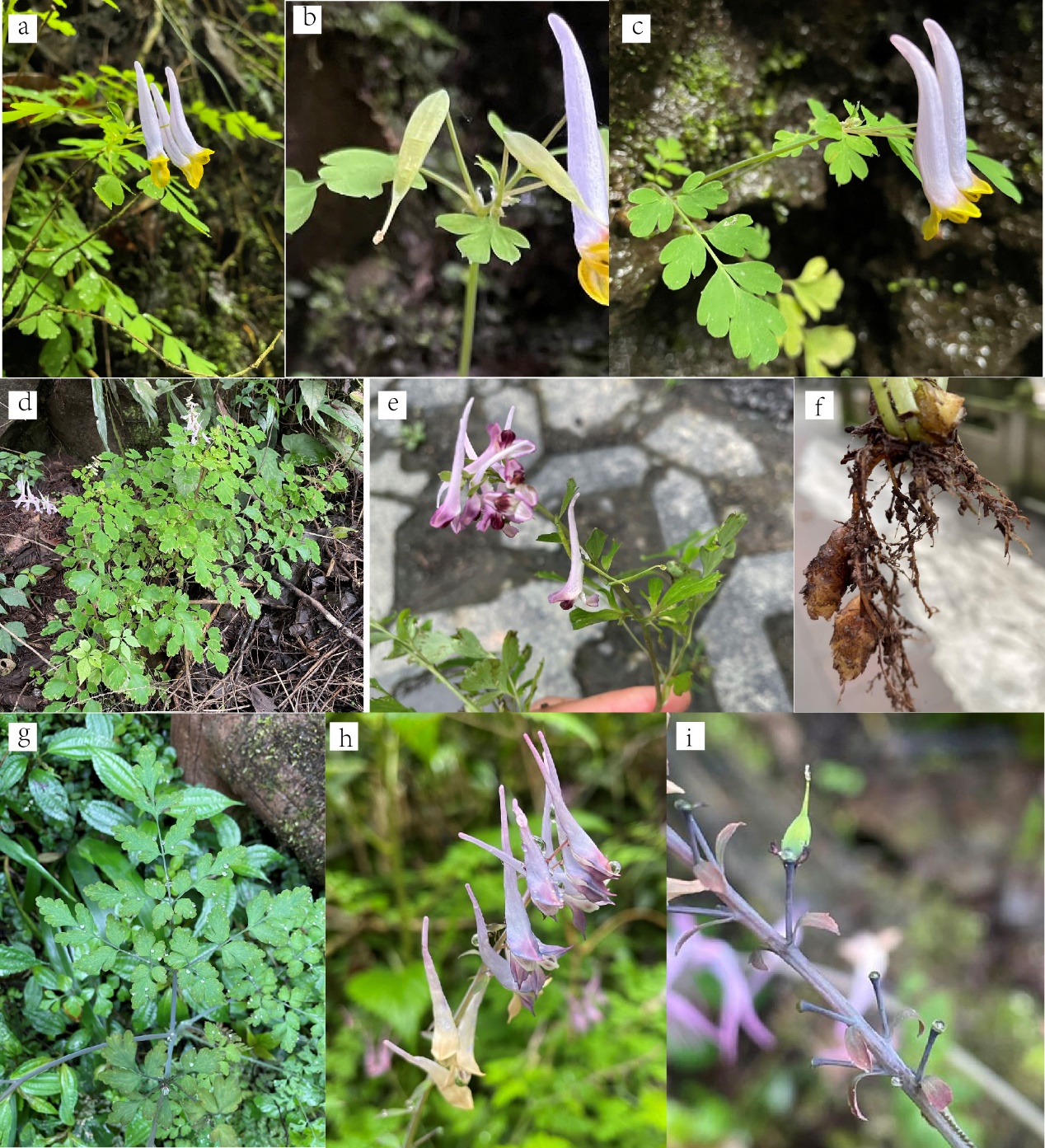


**Supplementary figure 1** Field pictures of three *Corydalis* species. a~c), the morphological characteristics of *C.* *pinnata (*herbarium specimen number: CDCM0005285*); d~f),* the morphological characteristics of *C. sheareri,(* herbarium specimen number:CDCM0005283*); g~i), t*he morphological characteristics of *C. mucronata,(* herbarium specimen number: CDCM0005284*).*
